# Determining Core Components in Accreditation of Limited Surgery Facilities in Iran

**DOI:** 10.31661/gmj.v9i0.1729

**Published:** 2020-06-25

**Authors:** Nader Asgari, Somayeh Hessam, Irvan Masoudi Asl, Shaghayegh Vahdat

**Affiliations:** ^1^Department of Health Services Administration, South Tehran Branch, Islamic Azad University, Tehran, Iran; ^2^Department of Health Services Management, School of Management and Information, Iran University of Medical Sciences, Tehran, Iran

**Keywords:** Day Care Centers, Accreditation, Comparative Study

## Abstract

**Background:**

Limited surgery facilities, or day-care centers, have been expanding in recent years with the approach of reducing the number of patients referred to hospitals for treatment in relation to limited and ambulatory surgeries. This study was conducted to perform a comparative review of accreditation models for limited surgery facilities of selected countries and to obtain expert opinions in the field of policymaking and accreditation in Iran.

**Materials and Methods:**

This applied and qualitative study was carried out by a comparative method in 2019. The accreditation standards of limited surgery facilities in nine selected countries/states were assessed. Semi-structured interviews were then held with 25 Iranian experts in policymaking as well as accreditation authorities.

**Results:**

Evaluation of the core components of accreditation standards for limited surgery facilities in selected countries showed that the main concepts of care and treatment, human resource management, patient safety, drug management, patient education, health information management, quality improvement, service recipient rights, infection prevention and control, physical structure, management and leadership, and general facilities were among the key recurring concepts in all models. In the study of factors affecting the accreditation model of limited surgery facilities in Iran, 5 main topics and 43 subtopics were identified.

**Conclusion:**

Although the current assessment model of limited surgery facilities is an appropriate tool for evaluation, it still needs to be improved because of the uncertainty of evaluation model, training of accreditors and the content of standards.

## Introduction

All public and private organizations need performance appraisal and quality assessment systems to measure the efficiency and effectiveness of their human resources in order to achieve sustainable development and growth in today’s competitive arena [1]. To attain this goal, accreditation standards have been implemented with a focus on clinical and non-clinical services [2-4]. Among health care providers, limited surgery facilities have been growing in recent years, playing a role in the reduction of patient referral to hospitals with respect to limited and ambulatory services. A limited and ambulatory surgery facility is an institution the patient can leave within a few hours (>24 hours) after surgery [5-7]. Designing an accreditation model to ensure the quality of limited surgery facilities appears to be necessary to ensure community health and prevent unexpected deaths considering the importance of quality in performance of limited surgery facilities with respect to surgical interventions in health care, high expense of operation, large number of patients and clients [8,9]. In Iran, local accreditation standards of hospitals have been developed and implemented since 2012, which are being extended to other health care providers Finally, national checklists were issued in 2018 to supervise limited and ambulatory surgical facilities in Iran. Comparative studies on the authorization of limited surgery facilities are a prerequisite for the development of accreditation standards. Although several researches have been carried out around the world to evaluate the anticipated conditions of medical services to offer services in limited surgery facilities and various conceptual models have been offered for their management, few studies have been done regarding the accreditation of limited surgery facilities in Iran. The goal of this study was to assess the accreditation standards of limited surgery facilities in selected developed countries and to identify the factors influencing the accreditation model of limited surgery facilities in Iran according to the opinion of experts in this field.

## Materials and Methods

The present research was an applied study that was qualitatively performed in two phases: comparative survey and interview with experts in 2019 [10]. For this purpose, a comparative review was first performed on the research population, namely limited surgery facilities of selected countries having an effective accreditation system, as well as countries with a similar situation to Iran that have implemented a successful system. The samples were taken from websites of official organizations active in the field of health as well as related centers with accreditation standards of limited surgery facilities in certain countries. The research sample was chosen from designated countries and models to be included based on the index of being a forerunner and having the longest historical background in accreditation, and national and international models from USA and Canada were thus preferred. Moreover, a sample from Asia (United Arab Emirates) and certain models from Europe and Australia were included in this study for further comprehensiveness. Finally, the accreditation standards of limited surgery facilities in nine developed countries/states, including six US states (International Joint Commission [JCI], Mississippi State, American Association for Accreditation of Ambulatory Surgery Facilities [AAAASF]), Canada (including Accreditation Canada International [ACI] and the state of Alberta), Australia, Spain, United Arab Emirates, and Iran were reviewed. Data extraction form was used to collect data from nominated countries and comparative tables were applied to analyze and compare the data. Accreditation comparison of limited surgery facilities in countries of choice focused on various domains such as basics and principles of accreditation, major and minor components, the implementation process, and the legal requirements and ratings. Subsequently, a qualitative study was conducted using the Framework Analysis method aimed at holding interviews with experts of policymaking and accreditation in Iran. Hence, the assessors and chief surveyors were selected by snowballing. Accordingly, 25 accreditation experts, authorities and assessors were interviewed by formal invitation. In-depth interviews were designed [11] and semi-structured interviews were held to collect the data according to a guideline. A member of the research team held all the interviews at the workplace of interviewees, which lasted on average 50-60 minutes and additional interviews were conducted if necessary. Sampling continued until data saturation. In the results section, the letter “M” attached to a number means a quote from an interviewee. The main construct of the interview was the identification of key dimensions affecting content, assessors, and accreditation method of limited surgery facilities. Lincoln and Guba’s trustworthiness criteria, which are equivalent to validity and reliability in quantitative research were used to achieve data validity and reliability. To this end, four criteria of credibility, dependability, confirmability, and transferability [12] were evaluated. The researcher attempted to satisfy the criteria through the careful choice of key informants, long-term contact with stakeholders and winning their trust, integration of data collection methods (such as interviewing, field notes, reminder writing), triangulation of different policymakers in the field of accreditation, use of interview guides, allocation of sufficient time for interviews, ongoing review and continuous comparison of data and classes in terms of similarities and differences, re-checking findings with participants, providing detailed and in-depth data analysis as well as research-rich descriptions for readers. Prior to each interview, informed consent was obtained from participants to enter the study, with an emphasis on privacy, confidentiality, and correct disclosure of information without mentioning the names. To analyze the data, Framework Analysis was used, which consists of five steps; familiarization, identifying a thematic framework, indexing, charting, mapping, and interpretation [13]. This approach is specifically designed to analyze qualitative data for policymaking purposes. During the familiarization phase, a communication-content summary was designed for each interview. Initial thematic framework was designed according to research literature, interview guide questions, and thematic instructions [14]. The framework was discussed in several sessions with research team members, which was revised through reviewing interviews and repeating the familiarization phase. Various sections of the interview data were indexed by subject matter through one or more codes, which were repeatedly reviewed and modified by the research team and discussed for the last time in a session by research members. We compared the viewpoints of interviewees on each subject with the help of analytic tables. The relationship between topics and subtopics was also identified and analyzed. The transcribed interviews were consulted wherever necessary and supplemented with analysis tables if needed. The next step was the interpretation of topics, for which a process similar to that described in the indexing step was performed [14]. The thematic framework was frequently reviewed and upgraded throughout the analysis process [15]. The concepts, contradictions, theories, experiences, and researches were also compared with each other and the desired patterns and relationships were deduced from the findings. The coding process began when data were collected and basic concepts identified in the form of initial codes. Afterward, the codes with the same concept were sequenced to form subtopics. MAXQDA 10 software (Germany) was used to manage the data and the baseline framework had four themes that were increased to five during the analysis.

## Results

###  Comparative findings

The comparative findings of this research include the type and number of major and minor components of the standard as well as the accreditation method of surgical centers in selected models. Table-1 separately lists the core components in designated accreditation models of surgical centers. The comparative results on the content of standards indicate common concepts at the level of core care and treatment components, human resources management (HRM), patient safety, drug management, patient training, health information management, quality improvement, service recipient rights, infection prevention and control, physical structure, management and leadership, and public facilities in the four models. Furthermore, the concepts of environmental health, medical equipment management, disaster risk management, paraclinical departments, access to and continuity of care, patient transfer, nursing care, dentistry department, patient identification, blood and blood products, prevention of patient’s fainting, support services, statutory licenses and compliance with legal tariffs had the lowest degree of sharing at the level of core components (Table-2). Functional and departmental models are employed to formulate the standards and some countries have used a combination of the two models. In the functional model, themes such as patient rights, access to care, patient evaluation and safety, management and leadership, facility management, etc. are considered in relation to hospital management issues. In the departmental model, separate standards are developed for clinical, paraclinical, support and management departments [8]. With respect to procedure and assessors of accreditation for surgical facilities in nominated models, the accreditation body in JCI, AAAASF, ACI, Alberta, and Australia models is an independent accreditation organization and it is the Iranian Ministry of Health and Medical Sciences (MOHME) in other models. In all the models reviewed, metrics of higher importance and weight were mentioned as a requirement or prerequisite for the accreditation process. Certificate validity period varied from one year (in the Mississippi model) to four years (in ACI, Alberta and Australian models, Table-3).

###  Evaluation of expert viewpoints 

 In this study, 25 experts were interviewed about the factors affecting accreditation models of limited surgery facilities in Iran. Finally, 5 main topics and 43 subtopics were categorized through a review of subject matters. The main topics were (1) content of accreditation bases of limited surgery facilities, (2) development of standards, (3) role of assessor (4), accreditation method, and (5) overall scoring (Table-4). In this section, we discuss how the main topics and sub-topics are formed by quoting from interviewees. In interviews with experts, attention to physical structure; management, leadership and human resources in the facility; admission process; discharge and continuity of care and treatment (before, during and after surgery); anesthesia and surgical complications; patient training and safety; control and prevention of infections; waste management; medical records of patients; provision of services and paraclinic; drug and equipment; patient rights and relationship between facility and auxiliary hospital were identified as minor and key issues. “Components that represent the existence of an organization should be considered as the key themes and objectives of an organization as standards and metrics” (M 23). “The physical structure and facilities needed to provide services to patients in compliance with the principles of safety and hygiene are important prerequisites for acceptable quality and services at limited surgery facilities” (M 6). “Given that limited surgery facilities are daycare centers smaller than a hospital, management and leadership issues are not highlighted and there is a higher emphasis on patient and safety management of the facility” (M 3). “Management and leadership does not require a complex organization but must be addressed in the areas of human resources management, risk management, and patient safety” (M 6). “HRM is highly influential in terms of patient safety in limited surgery facilities from the perspective of recruiting authorized and qualified staff. Obviously, other issues such as empowerment and evaluation of staff are also important. However, the technical and legal competence of personnel, especially clinical staff, surgeons sand anesthesiologists is of high importance”(M9). “In a procedural view to limited surgery facilities, patient admission and evaluation of general conditions, surgical and anesthetic care, post-surgical care until patient’s discharge as well as safe discharge and follow-up should be assessed for accreditation” (M 18). Limited surgery facilities provide service to patients and discharge them within 24 hours, and continued care and treatment from the moment of admission to full recovery of the patient must be planned and carefully evaluated for accreditation (M 12). “Surgical facilities in Iran perform a defined range of surgeries; therefore, patient admission and general conditions should be regarded as a key component” (M 1). “Complications after surgery and even following the discharge of patients are an important component patients provision of safety services at surgical facilities, so prevention and management of these complications is a key issue leading to the safe discharge of patients “ (M 5). “Given the importance of post-surgical patient follow-up in terms of possible complications and infections as well as subsequent visits for dressing and continuing treatment, the surgical facility should carefully and systematically perform the post-discharge follow-up procedure that must be assessed in the accreditation system for such facilities” (M11). “A surgical facility must adhere strictly to all safety standards and principles in environmental and human aspects and other features from the admission of the patients until their safe discharge, as the patient will not be under long-term care after surgery and will be discharged” (M 8). “Effective communication with patients along with proper and complete training should be considered because of the short stay at the facility and the necessity of participation in the continued treatment process, and the surgical center should know what training is required for each patient and how this relationship will be continued (M 10).”Prevention and control of infection as one of the most common surgical complications is of importance and the surgical facility must have a specific plan in place”. (M4). “Surgical facilities are sporadically distributed in a city and because their medical waste can be hazardous to community health, the observation of environmental health and management of medical waste at these facilities should be assessed in the accreditation of the centers, which should be held responsible for safe disposal of dangerous waste” (M14). “Medical information is not properly recorded at surgical centers, and patients do not have access to their clinical records in case of any problem. Therefore, we recommend a theme by the name of completing patients’ medical records” (M 7). “Paraclinic services such as laboratory tests and imaging facilities should be provided according to the type of patient and surgery as well as in critical conditions of patients, and patients’ needs for paraclinical services are components for accreditation of the facility” (M13). “The surgical facility must supply, maintain, and use medical equipment properly, which is particularly important for anesthesia and operating room equipment” (7). “Since surgery centers offer service privately outside the hospital environment, the management of critically ill patients should be considered in specific cases. It is essential that the center cooperates with an auxiliary hospital to support critically morbid patients and evaluate the way in which acute and critically ill patients are transferred and cared for” (M 2). A majority of interviewees believed that one can use a hospital model as the basic approach to formulating the core accreditation components of limited surgery facilities in Iran because it is the first year of implementation and that the provision of health care services is similar to a small-scale hospital. “The accreditation in the field of limited surgery should normally use the experience of hospitals but we must consider the nature and mission of limited surgery facilities in formulating the themes. On the other hand, since service recipients are the same in hospitals and limited surgery facilities, there should be no overall difference in main components; however, we are required to address the specific differences and missions of limited surgery facilities in the form and content of themes” (M 24).” Considering that these facilities are to be accredited for the first time in Iran, the standards should be developed in a way to be understood, processed and easily implemented. Obviously, it will be possible to expand the standards in future reviews” (M 15). In my opinion, we must not regard these facilities as separate from the healthcare system because a limited surgery facility is where healthcare services are presented similar to a small hospital, and a number of hospital standards can be omitted and some specific standards added to finalize the content of their standards” (M 23).” The literature and development mode of standards for surgery facilities should not be different from hospital standards because many medical practitioners, nurses and other staff are employed in both facilities and have been associated with accreditation literature in hospitals for many years” (M 17). In exploring the opinions of experts and authorities, paying attention to structural and process standards, utilizing the technical knowledge of surgeons and anesthesiologists, the involvement of all stakeholders, applicability, ease of understanding, and a functional approach in formulating accreditation standards were recognized as other subtopics. “Development of standards has to be functional, and a sectarian look to quality must be avoided. Process and infrastructure standards should receive particular attention as they are implemented in the first year” (19). Because it is the first standards implementation year of limited surgery facilities, the standards need to be expressed in a simple and understandable way and to take full advantage of them, the involvement of specialized groups in the surgical and anesthesiological associations as well as other key stakeholders must be maximized “(M22). Because assessors are a key component of the accreditation process and final judgments and decisions of them directly affect the accreditation outcome, the interviewees believed that evaluators of limited surgery facilities must have prior assessment experience, relevant education, and clinical information as well as work experience in the operating room and limited surgery facilities. “Assessors ought to be selected from among experienced clinical staff and preferably from operating room personnel and must complete additional training” (M 22). “The assessors must have work experience in the operating room, be associated with operating room specialists, surgeons, and other clinical disciplines. On the other hand, with regard to comparable missions of limited surgery facilities and hospitals, accreditation assessors of limited surgery facilities must have the technical capability and general skills similar to hospital assessors” (M 23). According to the interviewees, it is preferable for limited surgery facilities to be accredited by an independent evaluating agency without any affiliation with MOHME; however, because we are in early implementation years of certification, the accreditation has to be mandatory and valid for less than two years (at least within the first years after announcing the standards). “Until reaching a level of maturity in the country when an independent accreditation body conducts an assessment, mandatory accreditation of limited surgery facilities should be undertaken by MOHME as defined and required by the Health System Act of Iran” (M 16). “Given that the standards of limited surgery facilities are in their early years of implementation, there is a need for two separate assessments to be conducted within one year or to give certification in a one-year period, which can be increased two years afterward” (M 21). “Considering that limited surgery facilities are like a small hospital, it is inappropriate for them to be accredited for an interval shorter than a hospital, and even with respect to the type of patients admitted, it is better for such facilities to be monitored and evaluated even more stringently than a hospital (M 23). According to the interviewees, there should be similar procedures to the hospital accreditation model in terms of processes such as self-assessment and weighting of standards. Moreover, in the early years of implementation, there would be no weighting and classification of standards. “Considering the structural similarity and presentation of therapeutic and clinical services comparable to hospitals at limited surgery facilities, I see no difference in the type of quantitative assessment and scoring between these centers and hospitals” (M 24). “Measures should not be classified or weighted in the first round and the self-assessment process could be included in the accreditation program” (M 21). Interviewees also believed that to be effective, limited surgery facilities would need to obtain legal permits prior to the beginning of the accreditation process. According to experts, the subtopics addressing the rating of limited surgery facilities include ranking of centers based on average score in the first year, the direct and indirect impact of rating results on center’s earnings, classification of permitted practices according to accreditation ratings, as well as the effect of potential violation records on accreditation scores of facilities. If we rate-limited surgery centers, service recipients have more options for the type of facilities and can choose their preferred facility based on the tariff they will pay (M2). “Regarding that all the limited surgery facilities in the country are privately owned with the aim of having more profit, we will have stronger supervision and quality of service if the facilities are accredited in relation to their income” (M 23). “If the ranking of the facilities affects their income and service tariffs, they will be highly motivated to qualify their services” (M 20). “Since responding to non-medical needs in limited surgery facilities is more appropriate and satisfactory than public centers because of their privacy, it is better to rank these facilities on the basis of quantitative scores that will affect their earnings. In fact, anything that affects the credibility of limited surgery facilities will have an impact on their income. Another option is that the accreditation scores of these facilities could have an impact on their list of permitted practices (24). “It would be much more effective if supervision were to be taken into account in the accreditation of limited surgery facilities. In our managerial experiences, supervisory practices have been more effective in dealing with violations in limited surgery facilities than hospitals; indeed, these centers have more concerns than hospitals. It is suggested that the records of supervisory notes in the face of potential violations be taken into account in the ranking of limited surgery facilities” (M 22).

## Discussion

Accreditation is defined as the process of self-assessment and external evaluation by health care organizations to properly assess performance in relation to developed standards and implementation methods for continuous improvement [16,17]. In this study, the accreditation models of limited surgery facilities in a number of developed countries were assessed and contrasted with the existing model of Iran in a comparative study. Then, by interviewing Iranian experts and authorities in the field of policymaking and accreditation, factors affecting the design of an accreditation model for limited surgery facilities in Iran were identified. In a comparative study of accreditation models in the world, it was found that the current local and executive model in Iran is not in accordance with the structure of the health system and provision mode of limited surgery services for accreditation of these facilities and that it does not have the required efficiency and effectiveness. The current model differs from other countries in terms of focusing on regulatory tools, including the requirement to obtain legal licenses to operate, comply with laws and regulations as well as legal tariffs. Although qualitative aspects such as care and treatment, human resources, patient education, prevention and hygiene, medicine and equipment, and service recipient rights are also found at the level of core components of this model, the study of subcomponents indicates that a supervisory approach and a checklist tool have been emphasized to investigate the function of limited surgery facilities in the country. Various studies have found that hospital service accreditation is not the only approach to qualify health care services and that there are other methods such as ISO, total quality management, six sigma, and so forth [18]. In a 2008 study, Sekimoto examined the impact of accreditation on infection control programs in Japanese teaching hospitals and showed that hospital accreditation had a significant impact on the infrastructure and performance of infection control programs in educational hospitals [3]. Similar results have been observed in other scientists’ reviews regarding the positive impact of accreditation on the improvement of service quality [19-24]. Other points to be improved in the Iranian model compared to other international models regarding additional accreditation aspects of limited surgery facilities include the assessment by MOHME and the lack of quality rating following obtaining the assessment results. In 2011, Chaghari & Ameriyoun discussed the challenges of hospital accreditation process in the Islamic Republic of Iran and noted that the most effective way to organize the assessment and accreditation of health care system in the country was the establishment of an independent institution with general title of “national accreditation center to Iranian health care services” [25]. Similar results were also noted in the study of Ameriyoun *et al*. in 2013 [26]. Therefore, there is a need to redesign the accreditation model of limited surgery centers in Iran. In the evaluation of the factors affecting the accreditation model of limited surgery facilities in Iran, five main accreditation topics of limited surgery facilities, standard development model, the role of the assessor, accreditation method and overall scoring, as well as 43 sub-topics, were identified. In terms of the content of accreditation topics, 20 subtopics were detected, which corresponded to the themes mentioned in the international models (Table-1). The results showed that paying attention to the hospital model in formulating the main themes, utilizing the technical knowledge of surgeons and anesthesiologists, the involvement of all stakeholders, simplicity and functional approach in formulating accreditation standards were the minor factors. Assessors should have previous evaluation experience, relevant education, and clinical information as well as a work record in the operating room and limited surgery facilities. The assessment was recommended by MOHME as compulsory within a period of fewer than two years in the early years of implementation, followed by an evaluation by an independent assessment agency in the coming years. In terms of overall scoring, the impact of ranking results on the income of a center and the level of permitted practices were extracted. The results of the qualitative study of Karimi *et al*. in 2013 detected 10 main topics and 72 subtopics in relation to the effect of hospital accreditation on service delivery from the viewpoint of experts. Their results show that accreditation is properly implemented with preparing infrastructure, correct selection of accreditation model, stakeholder justification concerning the need for accreditation, continuous monitoring, creation of appropriate information systems, transparency of information by changing the overall attitude of the organization, which has positive outcomes in achieving hospital goals and improving the quality of service [27].

## Conclusion

 From the study of international models and interviews with the help of experts, it can be concluded that the current accreditation model of limited surgery facilities is insufficient and needs to be seriously reviewed. This type of review is suggested in three areas; the content of the standards, method of assessment, and evaluation of assessors. In terms of the content of standards, the existing model is not comprehensive and does not cover all functional aspects of surgical facilities. According to the evaluation and recruitment approach of assessors, the current model is not adequate from the viewpoint of experts and is a mere supervisory aspect observing the laws and regulations. The existing model is mainly self-evaluative and does not provide any credible output that can improve the quality and promote services. The ranking of centers is not addressed but implemented by inspectors of MOHME and medical universities not undergoing training courses with a supervisory approach without any regard to the basics of quality promotion. Therefore, it is strongly recommended to rate-limited surgery facilities following a qualitative evaluation. Experienced clinical assessors with sufficient experience and work record in operating rooms who have passed accreditation training must be employed. Regarding the content of standards, it is also suggested that with regard to the first accreditation round of these centers, the standards should be reviewed with simple concepts, prioritizing the process and placing more emphasis on patient safety management.

## Conflict of Interest

 There is no conflict of interest in this study.

**Table 1:** Comparison of core components under study in different countries

**Row**	**Main components under study**	**Country/State**	**References**
1	International Patient Safety Standards, Access to and Continuity of Care, Patient and Family Rights, Patient and Family Assessment and Care, Anesthesia and Surgery Care, Drug Management and Use, Patient and Family Training, Quality Improvement, Infection Prevention and Control, Governance, Management & Leadership, Facility & Safety Management, Staff Training & Qualification & Information Management	Joint international commission (JCI)	[28]
2	Executive Management, Organizations and Personnel, Policies and Processes, Existence of Medical Personnel Organization, Patient Transfer, Safety, Housekeeping (Service), Laundry, Cleaning, Equipment Maintenance, Crisis Management, Medical Records System, Nursing Care, Surgery, Anesthesia, Dentistry, Environmental Health, Central Sterilization, Pharmaceutical Services, Radiology Services, Laboratory Services, Medical Facilities, Public Facilities and Disaster Preparedness Program	US state of Mississippi	[29]
3	Main Missions, Policies and Policies & Operating Room Environment, Post-Anesthesia Care Unit, Public Safety at the Center, Medicines and Liquids, Medical Records, Quality Improvement, Staff and Anesthesia	American Association for Accreditation of Ambulatory Surgery Facilities (AAAASF (	[30]
4	Buildings with Effective Facilities, Establishment of a Safe Center, Appropriate Personnel to Provide Services, Surgical/Medical Services, Patient Information Maintenance, Safety Monitoring and Quality of Surgical/Medical Services	Accreditation Canada International (ACI)	[31]
5	Staff, Patient Care, Infection Prevention and Control, Facilities, Supplies & Equipment, Documents and Forms, Safety Standards, Quality Assurance and Improvement	Canadian state of Alberta	[32]
6	Patient Care, Diagnostic Services, Pharmaceutical and Drug Administration Management, Support Services, Safety, Patient and Family Rights, Health Information Management, Administrative and Human Resources Standards, Supply and Facility Management	UAE	[33]
7	Managing and Governing Safety and Quality of Service Provider Organization, Engagement with Clients (Recipients), Preventing and Controlling Health-related infections, Medication Safety, Patient Identification and Compliance of Therapeutic Approaches, Clinical Guidance, Blood and Blood products, Prevention and Management of Surgical Injuries, Identification and Response to Medical Risks in Acute Surgical Patients and Prevention of Patients Fall and Resulting Injuries	Australia	[34]
8	Recipient Rights, Patient Safety, Organization and Management, Physical Structure and Resources, Human Resources and Quality improvement	Spain	[35]
9	Licensing, Human Resources and Law & Documentation, Care & Treatment, Training, Medical Information & Documentation, Physical Space, Prevention & Health, Drugs & Equipment, Observers, Tariffs	Iran	[36]

**Table 2:** Comparison of key concepts at the level of core components in examined models of designated countries

**Repeated cases**	**Number of repeated cases**	**Common concept at the level of core components**
American JCI model, Canadian ACI model, State of Alberta, State of Mississippi, AAAASF state and Australia, Dubai and Iran	8	Care & Treatment
American JCI model, Canadian ACI model, State of Alberta, State of Mississippi, AAAASF state and Spain, Dubai and Iran	8	HRM
American JCI model, Canadian ACI model, State of Alberta, State of Mississippi, AAAASF state and Spain and Dubai	7	Patient Safety
American JCI model, State of Mississippi, AAAASF state and Australia, Dubai and Iran	6	Drug Management
American JCI model, State of Mississippi, AAAASF state and Australia, Dubai and Iran	6	Patient Training
American JCI model, Canadian ACI model, State of Mississippi, AAAASF state and Dubai and Iran	6	Health Information Management
American JCI model, State of Mississippi, State of Alberta, AAAASF state and Spain and Dubai	6	Improvement of Quality
American JCI model, Australia, Spain, Dubai and Iran	5	Service Recipient Rights
American JCI model, State of Mississippi, State of Alberta, Australia and Iran	5	Infection Control & Prevention
Canadian ACI model, State of Mississippi, Spain and Iran	4	Physical Structure
American JCI model, State of Mississippi, Australia and Spain	4	Leadership & Management
American JCI model, State of Mississippi, State of Alberta, Dubai	4	Public Facilities & Equipment
State of Mississippi and Iran	2	Environmental Health
State of Mississippi and State of Alberta	2	Medical Equipment Management
Canadian ACI model and state of Mississippi	2	Risk Management of Events & Catastrophes
State of Mississippi and Dubai	2	Paraclinic Departments
American JCI model	1	Access to and Continuity of Care
State of Mississippi	1	Patient Transfer
State of Mississippi	1	Nursing Care
State of Mississippi	1	Dentistry Department
Australia	1	Identification of Patient
Australia	1	Blood & Blood Products
Australia	1	Prevention of Patient Fall
Dubai	1	Support Services
Iran	1	Licensing
Iran	1	Observation of Legal Tariffs

**Table 3:** Comparison of selected countries in terms of different areas considered in accreditation process of limited surgery facilities

**Country/state** **Studied item**	**Iran**	**Spain**	**Dubai**	**Australia**	**Canadian state of Alberta**	**ACI model**	**US AAAASF state **	**US Mississippi state**	**JCI model**
Choice of core components	Structural-functional	Structural-functional	Functional	Functional	Structural-functional	Structural-functional	Functional-task	Partial-functional	Functional
Selection of minor components	Structural-functional	Structural-functional	Functional	Functional	Structural-functional	Structural-functional	Functional	Partial-functional	Functional
Executive accreditation body	MOHME	MOHME	MOHME	Independent accreditation organization	College of physicians and surgeons of Alberta	Independent accreditation organization	Independent accreditation organization	MOHME	Independent accreditation organization
Type and level of criterion (required, essential)	Standards related to the licensing of an institution’s activity are considered a prerequisite for evaluation	A number of criteria are required and indicate statutory requirements	Prior to operating as licensed, the limited surgery centers must be approved for safe and effective operation	Some of the standards are called core and are required and some are developmental and optional	Classification of measures is must (shall), recommended (should) and optional (may)	A number of measures are referred to as ROP, or required organizational procedures	Safety criteria have been marked with gold due to their importance	A number of structural metrics have been provided as a prerequisite for obtaining a permanent license.	A number of metrics have been put forward as part of the accreditation requirements
Accreditation process	Involves comprehensive and random assessment	Includes comprehensive assessment	Includes comprehensive assessment	Involves self-assessment, comprehensive assessment and periodic evaluation.	Includes self-assessment, comprehensive assessment, and compliance checking of physicians’ activities with existing laws as well as periodic assessments	Involves self-assessment and comprehensive assessment	Involves self-assessment and comprehensive assessment	License release process interferes with accreditation	Involves self-assessment, comprehensive assessment and periodic evaluation.
Referral to rules and regulations of license release	Yes	Yes	Yes	No	Yes	No	Not specified	Yes	No
Accreditation period	No	Two-year	Two-year	3-4 years	Four-year	Four-year	Three-year	Annual	Three-year

**Table 4:** Main topics and sub-topics regarding the factors affecting accreditation model of limited surgery facilities

**Theme**		**sub-Theme**
Content of accreditation themes of limited surgery centers	- Patient safety - Infection control and prevention - Environmental health and waste management - Patient rights and meeting their non-medical needs - Medical records of patients - Surgical and anesthetic complications - Drug and equipment Morbid and critical patients - Provision of services and paraclinic - Auxiliary hospital	- Physical structure - Management & Leadership - HRM - Patient admission - Safe discharge - Patient follow-up after discharge - Surgical and anesthesia care - Post-surgical and anesthesia care - General clinical care - Patient training and communication
Development of standards	- Paying attention to structural and process standards in a variety of main, support and management processes - Use of hospital accreditation experiences - Use of technical knowledge of surgeons and anesthesiologists in formulation of standards - Stakeholder Engagement - Understandability - Executability - Operability - Processing
Role of assessor	- Assessment experience - Education and clinical data - Work record in operation room and surgical centers
Accreditation method	- Mandatory-optional - Regulation by MOHME or private sector - Interval between two assessments - Self-evaluation process - Weighting - Grading of standards - Scoring of standards - Obtaining legal licenses based on bylaws as a prerequisite for accreditation
Overall scoring	- Ranking of centers based on overall average score in the first year- Direct and indirect impact of ranking results on center’s revenue - Classification of permitted practices based on accreditation rating of limited surgery centers - Influence of records from possible violations of centers on their accreditation
